# Comorbidities Between Specific Learning Disorders and Psychopathology in Elementary School Children in Germany

**DOI:** 10.3389/fpsyt.2020.00292

**Published:** 2020-04-28

**Authors:** Linda Visser, Julia Kalmar, Janosch Linkersdörfer, Ruth Görgen, Josefine Rothe, Marcus Hasselhorn, Gerd Schulte-Körne

**Affiliations:** ^1^Department of Education and Human Development, DIPF | Leibniz Institute for Research and Information in Education, Frankfurt am Main, Germany; ^2^Center for Research on Individual Development and Adaptive Education of Children at Risk (IDeA), Frankfurt am Main, Germany; ^3^Department of Child and Adolescent Psychiatry, Psychosomatic and Psychotherapy, Ludwig-Maximilian-University Munich, Munich, Germany; ^4^Center for Psychological Psychotherapy, University of Heidelberg, Heidelberg, Germany; ^5^Department of Child and Adolescent Psychiatry, Faculty of Medicine Carl Gustav Carus, Technical University Dresden, Dresden, Germany

**Keywords:** specific learning disorder, attention-deficit/hyperactivity disorder, depression, anxiety disorder, conduct disorder

## Abstract

Children with reading and/or spelling disorders have increased rates of behavioral and emotional problems and combinations of these. Some studies also find increased rates of attention-deficit/hyperactivity disorder (ADHD), conduct disorder, anxiety disorder, and depression. However, the comorbidities of, e.g., arithmetic disorders with ADHD, anxiety disorder, and depression have been addressed only rarely. The current study explored the probability of children with specific learning disorders (SLD) in reading, spelling, and/or arithmetic to also have anxiety disorder, depression, ADHD, and/or conduct disorder. The sample consisted of 3,014 German children from grades 3 and 4 (mean age 9;9 years) who completed tests assessing reading, spelling as well as arithmetic achievement and intelligence via a web-based application. Psychopathology was assessed using questionnaires filled in by the parents. In children with a SLD we found high rates of anxiety disorder (21%), depression (28%), ADHD (28%), and conduct disorder (22%). Children with SLD in multiple learning domains had a higher risk for psychopathology and had a broader spectrum of psychopathology than children with an isolated SLD. The results highlight the importance of screening for and diagnosing psychiatric comorbidities in children with SLD.

## Introduction

Children with specific learning disorders (SLD) do not only exhibit difficulties in reading, spelling, and/or arithmetic. They also often struggle with externalizing and internalizing problems such as attention deficits and hyperactivity, conduct problems, anxiety disorder, and depression (1). There is some evidence for the increased risk of symptoms and the diagnosis of attention-deficit/hyperactivity disorder (ADHD) in children with reading and/or spelling disorder (2–4). In a representative German sample of 2nd and 3rd graders, a comorbid ADHD diagnosis was found in 17.2% (isolated reading disorder), 20.3% (isolated spelling disorder), and 22.2% (combined reading and spelling disorder) of subjects with SLD (5). In contrast, in a general population sample in Germany only 5% of the 7- to 10-year old children met the criteria for ADHD (6); worldwide ADHD prevalence was estimated 3.5% (95% CI = 2.6%–4.5%) (7). In sum, ADHD has the highest comorbidity rate with reading and spelling disorder compared to other mental disorders (8).

Another frequently replicated result is the association between anxiety disorder and dyslexia (2, 4, 9). Carroll et al. (9) showed that anxiety disorders were more than twice as prevalent in children with dyslexia compared to children without dyslexia.

Results regarding the relationship between depression and dyslexia are ambiguous. While Goldston et al. (2) report an association between the two disorders (seemingly moderated by symptoms of inattention); Carroll et al. (9) did not find such an association. Finally, Willcutt et al. (4) reported higher rates of depression in children and adolescents with reading difficulties, independent of the presence of ADHD.

Likewise, no conclusive statement can be made regarding the comorbidity of conduct disorders and dyslexia. Although some studies showed elevated symptoms of conduct disorders in dyslexic children and adolescents, this relationship is assumed to be moderated by the simultaneous occurrence of ADHD (3).

The co-occurrence of dyscalculia and mental disorders is still poorly investigated. Willcutt et al. (4) reported that children and adolescents with dyscalculia were more likely to meet the criteria for ADHD, conduct disorders, anxiety disorder, and depression. However, the diagnosis of conduct disorder revealed to be fulfilled only by those children and adolescents with dyscalculia who also met the criteria for ADHD, indicating that the relationship between conduct disorder and dyscalculia is moderated by ADHD. A study investigating SLDs in representative school samples from 2nd to 6th grade in Brazil revealed an association between ADHD and dyscalculia (10). In contrast, Schuchardt et al. (5) did not find elevated rates of ADHD in children with dyscalculia. In a longitudinal study investigating the co-occurrence of internalizing symptoms (anxiety disorder, depression), children and adolescents with dyscalculia generally exhibited higher symptom levels than control subjects, although in the normal range (11). Similar to research results in dyslexic children, the elevation of anxiety scores in subjects with dyscalculia might be domain-specific (12). On the other hand, the aforementioned Brazilian study found an association between anxiety disorders and dyscalculia in 2nd to 6th graders (10). In a very recent meta-analytic evaluation, dyscalculia showed high comorbidity with ADHD symptoms (particularly inattention), as well as symptoms of the internalizing and externalizing spectrum (13). Taken together, ADHD also seems to play an important role with regard to dyscalculia. However, the relationship is not as well explored as in reading and/or spelling disorder.

ADHD does not only seem to have the highest comorbidity rate with SLD, it has also been suggested as a possible explanation for the comorbidity between SLD and other forms of psychopathology (4, 14). The strong association between ADHD and SLD could be partly explained by co-occurrence of different working memory deficits (15). More specifically, ADHD seems to be related to deficits in the central executive part of working memory, while dyslexia appears related mostly to deficits in the phonological loop, and dyscalculia to deficits in the visual-spatial sketchpad.

Subjects with deficits in more than one domain of academic achievement exhibit more psychopathological symptoms (4, 16). Thus, the worse children and adolescents perform academically, the more psychological distress they exhibit.

In the present study, we used a large non-clinical sample of 3rd and 4th grade children in Germany to shed more light upon the co-occurrence of different SLD subtypes and psychopathology. This is of great practical importance, as unidentified mental problems and mental disorders may impede treatment success in SLD. As opposed to earlier studies, we took into account various subtypes of SLD and various domains of psychopathology. Hence, not only the comorbidity between the various types of SLD and psychopathology could be studied, but also the comorbidities between the different types of psychopathology in children with SLD.

We explore the occurrence of anxiety disorder, depression, ADHD, and conduct disorder in children with an SLD in reading, spelling, arithmetic, or a combination of these. Additionally, we investigate to which extent the different psychopathologies co-occur within the different SLD groups. We hypothesize that children with reading and/or spelling and/or arithmetic disorder more often have depression, anxiety disorder, ADHD, and conduct disorder than children without SLD. The question regarding differences in the prevalence of psychopathological symptoms between the three types of SLD (reading, spelling, arithmetic) is explorative, as the literature is inconclusive. Also, the comorbidity between specific spelling disorder and psychopathological symptoms has been studied relatively infrequently (8). We expect that ADHD symptoms form one of the most common comorbidities in different types of SLD. We further expect that the more academic domains affected by SLD, the higher the risk for psychopathological symptoms, and the higher the number of areas in which a child, on average, exhibits psychopathology.

## Materials and Methods

### Recruitment

We invited families with 3rd and/or 4th grade children from the two German federal states Hesse (through the Hessian Ministry of Culture; n = 25,000) and Bavaria (addresses provided by local registration offices; n = 27,734) to participate in this study. The families were randomly chosen in a way that the population of selected families was approximately representative in terms of gender and age (8.8–10.8 years; Bavaria) respectively grade (Hesse).

Children and their parents were invited to download and use a web-based application to assess the academic skills and psychopathological profile of the children autonomously within 8 weeks. All parents and children gave informed consent. The study was approved by the ethics committees of the University Hospital of the Ludwig-Maximilians-University Munich and the DIPF | Leibniz Institute for Research and Information in Education, Frankfurt am Main. All subjects gave written informed consent in accordance with the Declaration of Helsinki.

### Participants

A total of 4,542 families started using the application (response rate 8.6%). Reasons for non-participation were not requested. After applying all filtering and exclusion criteria as described in the section below, the final sample consisted of 3,014 children with a mean age of 9;9 years (SD = 7 months; range 8;1–11;8). The sample is approximately equally distributed in terms of gender (1,570 [52.1%] boys and 1,444 [47.9%] girls) and grade (1,404 [46.6%] 3rd and 1,610 [53.4%] 4th grade). In Hesse, 636 (21.1%) families participated and in Bavaria 2,378 (78.9%). In both states, gender is roughly balanced per grade. Mothers with a high educational level, implying high socioeconomic status (SES), are overrepresented: 2,090 (69.3%) mothers had absolved gymnasium, the highest school certificate in Germany, as opposed to 42.9% in the population (17). The percentage with non-German nationality (177; 5.9%) is lower than what would be expected based on demographic data [10.5%; (18, 19)]. Native German speakers are slightly overrepresented (20).

### Drop-Out and Exclusion

We excluded cases for which the child did not complete all tests up to session four (678; 14.9%) or the parent did not complete all questionnaires (652; 14.4%). To avoid statistical dependence, we randomly excluded the data of one sibling per pair (n = 49). We excluded 81 (1.8%) cases because of an IQ ≤ 70 and 99 (2.2%) cases because the parents answered to an open question that the child had hearing or visual problems, neurological diseases, or chromosomal defects. In total, we excluded 1,528 (33.6%) cases, resulting in the final study sample of 3,014 children.

### Collection of Data

For this study, a software company transformed all standardized psychometric tests and questionnaires from their paper-pencil versions to an online tablet/smartphone version. Before using the online version for the current study, a preliminary version was piloted with 25 children and seven parents. On the basis of the observations we made and the feedback we received, minor adaptations were made, resulting in the final version used for the current study.

Participants worked on the tests and questionnaires independently at home. The web-based application contained clear instructions for which parts had to be completed by the parents and which parts by the child. Also, the detailed information material for study participants contained the instruction that children had to complete the tests and questionnaires independently. For the children, tests and questionnaires were grouped into sessions which had to be worked on for four separate days. Children were asked to complete an optional fifth session (they would get their reward also without doing this) which included a newly developed spelling test (not reported in this manuscript). Each session lasted 30–45 min. There was one session for the assessment of parent (or other caregiver) ratings.

### Measures

Reading achievement was assessed using the “Wuerzburger Silent Reading Test–Revised” [WLLP-R; (21) parallel-test reliability r = .93 for 3rd grade and r = .82 for 4th grade]. Children were presented with a series of written words and asked to select the corresponding image among four options within 5 min. Spelling performance was assessed using the long versions of the “Weingarten spelling test for basic vocabulary” [WRT 3+ for 3rd graders; (22) parallel-test reliability r > .91, and WRT4+ for 4th graders; (23) parallel-test reliability r > .90]. Children had to fill in the blanks of missing words using the correct spelling without a time limit. Arithmetic achievement was assessed using the computer-assisted “CODY math test” [CODY-M 2-4; (24)]. The CODY-M 2-4 includes nine subtests focusing on basic number processing (counting, magnitude comparisons), complex number processing (number dictation, number line, domino count comparison, missing numbers), counting skills (addition, subtraction, multiplication, placeholder tasks), and visuo-spatial working memory (a matrix memory span task). All scholastic achievement tests used are recommended by the German evidence-based practical guidelines for diagnosis and treatment in reading and/or spelling disorder (25) or dyscalculia (13).

Nonverbal intellectual ability was assessed using three of the four subtests (sequences of drawing, classifications, matrices) of the short version of the “Culture Fair Intelligence Test” [CFT 20-R; (26) test reliability r = .92]. The fourth subtest could not be adapted to an online version. Because it contains fewer items than the other subtests, i.e., it contributes less to the total raw score, and is often too difficult for children in the age range of our study, the resulting IQ-scores nevertheless form a good approximation of the intelligence of the children. The parental survey started with a questionnaire about family and child background containing questions about parental educational level and work, ethnic background and language, family history, children’s developmental problems, psychopathology, as well as learning (interventions), and learning problems and psychopathology in the family.

To assess children’s psychopathology, parents completed standardized rating scales for ADHD (FBB-ADHS; Cronbach’s α = .86–.94), conduct disorder (FBB-SSV; Cronbach’s α = .63–.93), and depression (FFB-DES; Cronbach’s α = .89) of the widely-used “Diagnostic System of Mental Disorders for Children and Adolescents–II” [DISYPS-II; (27)]. Anxiety disorder was assessed using the “Screen for Child Anxiety Related Emotional Disorders”, German version [SCARED-D; (28) Cronbach’s α = .91].

### Data Preparation

We used REDCap (29) for data management and R [(30) version 3.5.0] for data analysis. Data and analysis code are available on the Open Science Framework (https://osf.io/9mxp2/). We normalized all diagnostic tests used to the complete sample that used the web-based application. Also, as we were unable to directly monitor the participants’ behavior, we applied plausibility checks to ensure the quality of the data. For example, we excluded data with unrealistically long or short response times, thereby filtering out unreliable data to the best of our knowledge. In total, we excluded 540 (11.9%) cases because of implausible data. For more information about the norm development and plausibility checks, please see the **Supplementary Material**.

### Classification of SLDs and Psychopathology

As criterion to classify children as having an SLD, we used a z-score of ≤ −1.5 in the respective standardized test of academic achievement, following the recommendation by the Diagnostic and Statistical Manual of Mental Disorders, 5th edition [DSM-5; (31)]. As we could not assess other information indicating an elevated likelihood for SLD (e.g., clinical or qualitative information), in which case the DSM-5 recommends a cut-off of 1 SD, we classified children with a z-score of > −1 as not having an SLD and excluded children with a z-score of > −1.5 and ≤ −1, to ensure optimally distinct classifications. We decided to apply this procedure because the test results were based on online assessments, in which we did not have the chance to observe the children. By excluding borderline cases, the classification as having or not having an SLD is more reliable.

For the different analyses, we created four types of variables indicating SLD status. First, we created a categorical variable grouping children into eight categories that differentiated between isolated SLDs and all possible combinations of SLDs in the different domains. Children with z-scores of > −1 in all three domains (reading, spelling, arithmetic skills) were classified as having no disorder. Children with a z-score of ≤ −1.5 in one domain and z-scores of > −1 in the other two domains were classified as having an isolated SLD. Likewise, children with z-scores of ≤ −1.5 in two domains and a z-score of > −1 in the third domain, or z-scores of ≤ −1.5 in all three domains, were classified as having specific combinations of comorbid SLDs. As described above, children with a z-score of > −1.5 and ≤ −1 in at least one of the three learning domains were excluded. Second, for the inferential statistical analysis, we created four categorical variables that reflect SLD status more broadly. First, “any SLD” indicated whether a child had an SLD in any of the three domains or not. Children were classified as having any SLD if at least one of their reading, spelling, and/or arithmetic z-scores was ≤ −1.5, and as having no SLD if all three z-scores were > −1. Second, “reading disorder” categorized children as having any form (both isolated and non-isolated) of reading disorder, or not. The variable classified children as having an SLD in reading if their reading z-score was ≤ −1.5 and as having no SLD in reading if this z-score was > −1, independent from their test scores in the spelling and arithmetic domains. This variable was constructed in a similar way for “spelling disorder” and “arithmetic disorder”. Third, a count variable indicated the number of domains in which the child had an SLD (possible values: 0, 1, 2, or 3). For example, a child who was classified as having an SLD in reading and in spelling, but not in arithmetic, received a value of 2. Children with a z-score between > −1.5 and ≤ −1 in at least one of the three domains were excluded. Fourth, based on the count variable, we created a categorical variable indicating whether a child had “no SLD” (number of SLDs = 0), an “isolated SLD” (number of SLDs = 1), or “comorbid SLDs” (number of SLDs > 1).

In line with the cut-off of more than 1 SD used in the original DISYPS-II, we classified children as fulfilling the cut-off score for each of the disorders anxiety disorder, depression, ADHD, or conduct disorder when they had a z-score of ≥ 1 in the respective questionnaire. We created a categorical variable for each area of psychopathology that indicated whether a child had the respective psychopathology. Based on these four variables, we created an additional variable indicating the number of areas in which the cut-off score for a psychopathology was fulfilled (range: 0–4).

### Statistical Analyses

We used descriptive statistics to compare the children in the different SLD-groups with regard to the occurrence of anxiety disorder, depression, ADHD, and conduct disorder. To illustrate the overlap of the different psychopathologies in children with and without SLD, we used the visualization technique “UpSet” (32). We first compared the overlap of the different psychopathologies between children with and without SLD. In a second step, we compared the overlap between the different SLDs.

We used one-sided Fisher’s exact tests to test whether the occurrence of psychopathology in the respective areas was significantly increased in cases of SLD. We computed this test for each of the four areas of psychopathology and for the presence of SLD in general, as well as separately for reading, spelling, and arithmetic SLD. The odds ratio (OR) with 95% confidence intervals based on the adjusted inverse hyperbolic sine transformation procedure [with pseudo-frequencies ψ1 = 0.6 and ψ2 = 0.4; (33)] provided a measure of effect size.

To test the hypothesis that the more academic domains are affected, the higher the risk for psychopathology, we used a trend test based on the generalized linear model with logit link function (logistic regression) and the Wald test statistic [see (34)]. Additionally, we computed an estimate for the trend as OR and the associated 95% Wald confidence interval. For each of the four areas of psychopathology, we tested for a positive trend (i.e., one-sided test) in their occurrence over the levels no SLD, isolated SLD, and comorbid SLD. As post-hoc tests, we used one-sided Fisher’s exact tests.

To test the hypothesis that the number of psychopathological areas increases with the number of SLDs, we used a generalized linear model with log link function (Poisson regression), with the number of SLDs as predictor and the number of psychopathological areas as outcome variable.

For each of the hypotheses, we corrected for multiple testing by setting the false discovery rate (FDR) to .05 using the modified FDR procedure by Benjamini and Yekutieli (35).

## Results

### Correlations Between the Various Domains of SLD and Psychopathology

To get a first impression of how academic performance was related to symptoms of psychopathology, we calculated correlations between the reading, writing, and arithmetic scores and the symptom scores for depression, anxiety disorder, ADHD, and conduct disorder. The results are presented in **Table 1**. All correlations are significant on a .01-level after FDR-correction. The correlations between learning ability and psychopathological symptoms are negative for all domains. These negative correlations are strongest for ADHD-symptoms, although still moderate, followed by depressive symptoms. The table also shows moderate to high correlations between the various learning ability scores (.38–.45) and between those for psychopathological symptoms (.31–.59).

**Table 1:** Correlation coefficients between the learning ability scores in reading, spelling, and arithmetic, and the scores for psychopathological symptoms of ADHD, anxiety disorder, conduct disorder, and depression.

	Reading	Spelling	Arithmetic	ADHD	Anxiety disorder	Conduct disorder
Reading						
Spelling	0.45					
Arithmetic	0.39	0.38				
ADHD	−0.21	−0.27	−0.28			
Anxiety disorder	−0.07	−0.06	−0.16	0.35		
Conduct disorder	−0.08	−0.15	−0.14	0.58	0.31	
Depression	−0.17	−0.20	−0.23	0.59	0.56	0.54

ADHD, attention-deficit/hyperactivity disorder.

### Numbers and Percentages of Children With Psychopathology per SLD Group

An isolated spelling disorder occurs in 47 cases (1.6%) within the sample; an isolated reading disorder in 55 cases (1.8%), and arithmetic disorder in 56 cases (1.9%). Note that these do not include all children with the mentioned learning disorder, but only those with an isolated learning disorder in one domain, which explains why the percentages are lower than the to-be-expected 6.7%^1^. **Table 2** shows the numbers of children that were categorized into each of the eight SLD groups as well as the numbers within these groups for which the chosen cut-off for anxiety disorder, depression, conduct disorder, or ADHD was fulfilled. Note that the frequencies in the second column do not add up to the total sample size of 3,014 due to the fact that we excluded children with a z-score between −1 and −1.5 for this classification. We refer to the Table in the **Supplementary Material** for information about the average intelligence quotients as well as reading, spelling, and arithmetic T-scores per SLD group.

**Table 2:** Numbers and percentages of children with anxiety disorder, depression, conduct disorder, and ADHD in children with different types of SLD.

SLD group	Freq. (%)	ADHD		Anxiety disorder		Conduct disorder		Depression	
		Freq. (male/female)		Freq. (male/female)		Freq. (male/female)		Freq. (male/female)	
		[%]		[%]		[%]		[%]	
		Yes	No	Yes	No	Yes	No	Yes	No
No disorder	2079 (69%)	199 (113/86)	1880 (958/922)	263 (124/139)	1816 (947/869)	240 (136/104)	1839 (935/904)	215 (119/96)	1864 (952/912)
		[9.6%]	[90.4%]	[12.7%]	[87.3%]	[11.5%]	[88.5%]	[10.3%]	[89.7%]
Isolated reading disorder	55 (1.8%)	9 (4/5)	46 (32/14)	9 (6/3)	46 (30/16)	2 (1/1)	53 (35/18)	10 (7/3)	45 (29/16)
		[16.4%]	[83.6%]	[16.4%]	[83.6%]	[3.6%]	[96.4%]	[18.2%]	[81.8%]
Isolated spelling disorder	47 (1.6%)	11 (8/3)	36 (26/10)	8 (4/4)	39 (30/9)	11 (9/2)	36 (25/11)	8 (7/1)	39 (27/12)
		[23.4%]	[76.6%]	[17%]	[83%]	[23.4%]	[76.6%]	[17%]	[83%]
Isolated arithmetic disorder	56 (1.9%)	14 (6/8)	42 (17/25)	11 (4/7)	45 (19/26)	12 (6/6)	44 (17/27)	13 (6/7)	43 (17/26)
		[25%]	[75%]	[19.6%]	[80.4%]	[21.4%]	[78.6%]	[23.2%]	[76.8%]
Comorbid reading & spelling	25 (0.8%)	5 (4/1)	20 (17/3)	4 (2/2)	21 (19/2)	5 (5/0)	20 (16/4)	13 (11/2)	12 (10/2)
		[20%]	[80%]	[16%]	[84%]	[20%]	[80%]	[52%]	[48%]
Comorbid reading & arithmetic	12 (0.4%)	3 (0/3)	9 (4/5)	3 (0/3)	9 (4/5)	5 (1/4)	7 (3/4)	4 (1/3)	8 (3/5)
		[25%]	[75%]	[25%]	[75%]	[41.7%]	[58.3%]	[33.3%]	[66.7%]
Comorbid spelling & arithmetic	16 (0.5%)	7 (2/5)	9 (4/5)	4 (0/4)	12 (6/6)	5 (3/2)	11 (3/8)	6 (2/4)	10 (4/6)
		[43.8%]	[56.2%]	[25%]	[75%]	[31.2%]	[68.8%]	[37.5%]	[62.5%]
Comorbid reading, spelling, & arithmetic	17 (0.6%)	7 (4/3)	10 (6/4)	5 (3/2)	12 (7/5)	4 (3/1)	13 (7/6)	6 (4/2)	11 (6/5)
		[41.2%]	[58.8%]	[29.4%]	[70.6%]	[23.5%]	[76.5%]	[35.3%]	[64.7%]
Total SLD (any disorder)	400 (13.3%)	112 (58/54)	288 (174/114)	83 (39/44)	317 (193/124)	87 (52/35)	313 (180/133)	111 (66/45)	289 (166/123)
		[28%]	[72%]	[20.8%]	[79.2%]	[21.8%]	[78.2%]	[27.8%]	[72.2%]

ADHD, attention-deficit/hyperactivity disorder; SLD, specific learning disorders.

The individual group sizes are relatively small. Based on the descriptive statistics, the occurrence of psychopathology in all four areas seems higher in the seven SLD groups than in the group of children without SLD, except for the occurrence of conduct disorder in isolated reading disorder. The occurrence of psychopathology seems to be highest in children with SLDs in multiple areas. Remarkably high are the occurrence of depression in children with comorbid reading and spelling disorder (52%), conduct disorder in children with comorbid reading and arithmetic disorder (42%), and of ADHD in children with combined spelling and arithmetic disorder (44%). For children classified as having any SLD, the occurrence of comorbid psychopathology are 21% (anxiety disorder), 28% (depression), 28% (ADHD), and 22% (conduct disorder).

**Figure 1:**
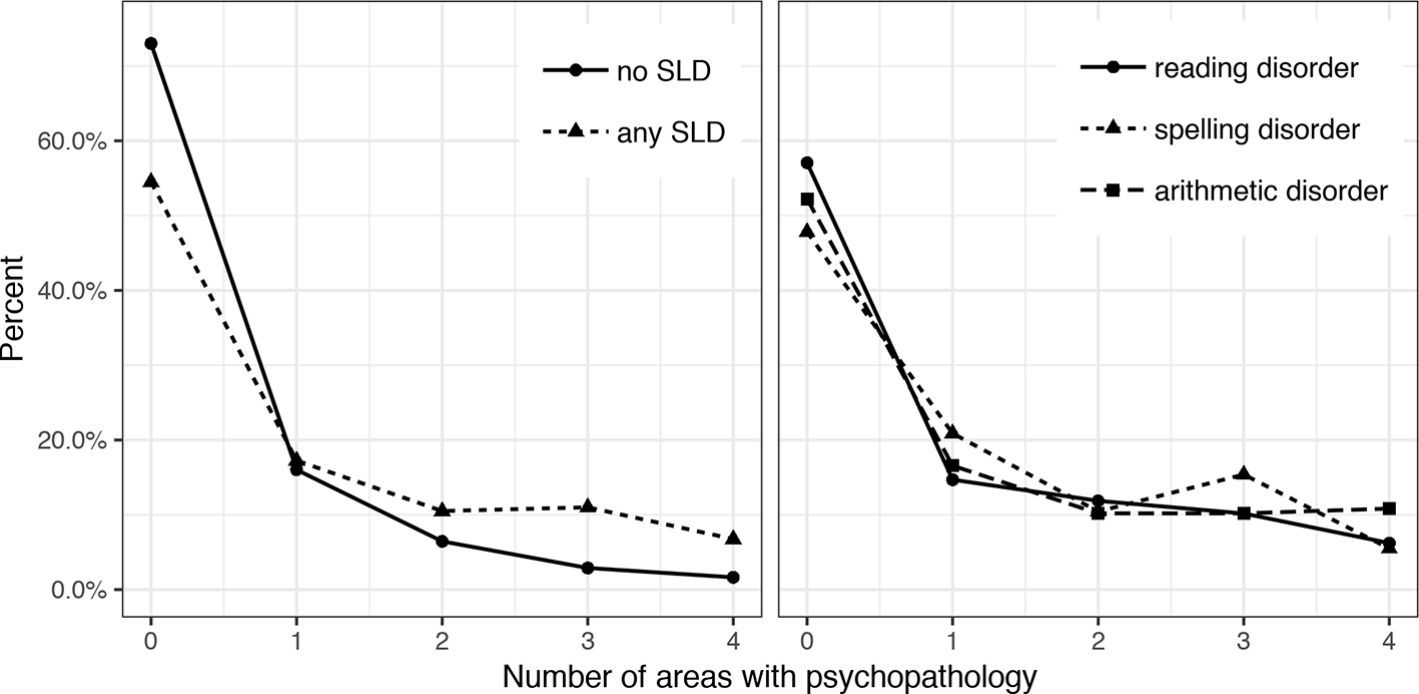
Number of areas affected by psychopathology in children with and without different subtypes of SLD, specific learning disorders (SLD).

**Figure 1** displays the number of areas with psychopathology for the more broadly defined groups of children with “any SLD” (n = 400), “reading disorder” (n = 177), “spelling disorder” (n = 182), and “arithmetic disorder” (n = 157). The percentage of children without any psychopathology is clearly lower in children with an SLD than in those without an SLD. While psychopathology in a single area occurs equally often in children with and without SLD, psychopathology in two or more areas occurs more often in children with SLD.

### Overlap in Psychopathology Within the SLD Groups

**Figure 2:**
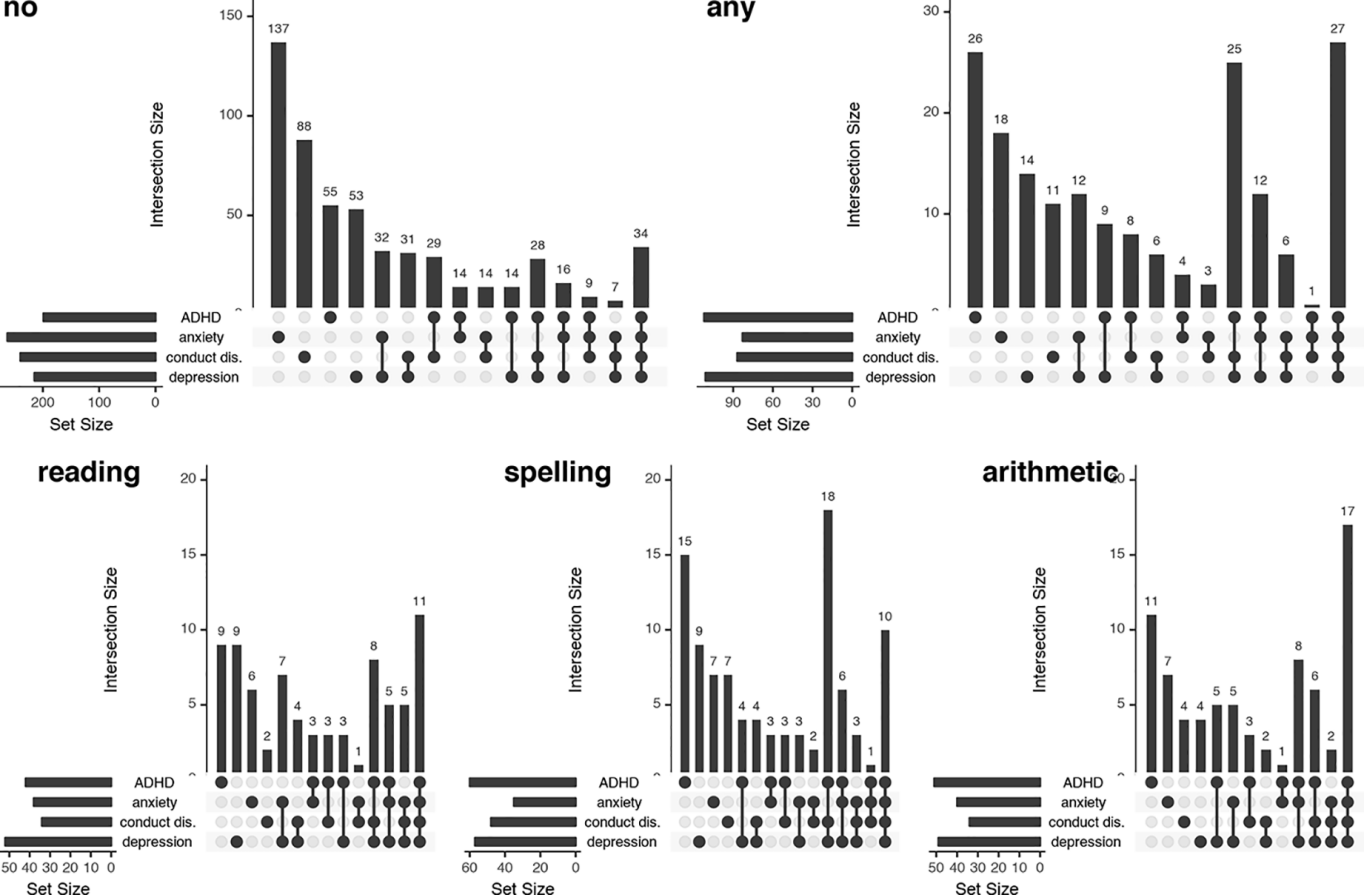
“UpSet” graphs visualizing the overlap between areas with psychopathology in children with no specific learning disorders (SLD), any SLD, reading disorder, spelling disorder, and arithmetic disorder. For each SLD group, the total number of children with the different psychopathologies [anxiety disorder, depression, conduct disorder (conduct dis.), and attention-deficit/hyperactivity disorder (ADHD)] is presented in the small horizontal graph on the left. In the graph on the right, the dots indicate the combinations of psychopathologies, and the bar above the respective dots indicates the number of children within this SLD-group affected by the respective psychopathologies. conduct dis., conduct disorder.

**Figure 2** illustrates this overlap between anxiety disorder, depression, conduct disorder, and ADHD. In children without SLD, anxiety disorder occurs most often, followed by conduct disorder. The graphs show that in children with SLD (note that there is overlap between these SLD-groups, because classification was done independent from the presence of a disorder in the other domains) there is a high degree of comorbidity between different psychopathologies. Within the group of children with any SLD, the highest rates occur for (1) ADHD only, (2) comorbid ADHD, depression, and conduct disorder, and (3) comorbidity of all four types of psychopathology. Group (3) is largest in cases of reading-related or arithmetic SLD as well. In children with spelling disorder, the largest group is formed by those with combined ADHD, depression, and conduct disorder, followed by the group with only ADHD. In children with reading disorder, depression occurs relatively frequently as well.

### Difference in Psychopathology Between Children With and Without SLD

**Table 3** shows the results of the Fisher’s exact tests of the association between SLD status and the presence of anxiety, depression, conduct disorder, and ADHD. The occurrence of all four different psychopathologies is significantly higher in children with than without SLD (p < .01). The odds of having ADHD are 3.67 (95% CI = 2.83–4.77) times higher if a child has an SLD. These odds are 3.33 (95% CI = 2.57–4.32) times higher for depression, 1.81 (95% CI = 1.38–2.38) times higher for anxiety disorder, and 2.13 (95% CI = 1.63–2.8) times higher for conduct disorder.

**Table 3:** Fisher’s exact test results for the difference in occurrence of anxiety disorder, depression, conduct disorder, and ADHD between children with and without SLD.

SLD	Psychopathology	p	OR (95% CI)
Any disorder	ADHD	<.001^*^	3.67 (2.83–4.77)
	Anxiety disorder	<.001^*^	1.81 (1.38–2.38)
	Conduct disorder	<.001^*^	2.13 (1.63–2.8)
	Depression	<.001^*^	3.33 (2.57–4.32)
Reading disorder	ADHD	<.001^*^	2.23 (1.57–3.24)
	Anxiety disorder	.007^*^	1.66 (1.15–2.43)
	Conduct disorder	.013^*^	1.62 (1.11–2.41)
	Depression	<.001^*^	2.93 (2.1–4.15)
Spelling disorder	ADHD	<.001^*^	3.81 (2.75–5.32)
	Anxiety disorder	.036	1.46 (1.01–2.17)
	Conduct disorder	<.001^*^	2.53 (1.8–3.61)
	Depression	<.001^*^	3.44 (2.48–4.82)
Arithmetic disorder	ADHD	<.001^*^	3.7 (2.61–5.28)
	Anxiety disorder	<.001^*^	2.26 (1.57–3.31)
	Conduct disorder	.001^*^	1.94 (1.33–2.91)
	Depression	<.001^*^	3.25 (2.29–4.65)

^*^Significant after FDR correction.

ADHD, attention-deficit/hyperactivity disorder; SLD, specific learning disorders.

When looking separately at reading, spelling, and arithmetic disorder, psychopathology is also elevated, except for anxiety disorder in children with spelling disorder, which cannot be considered as significant after FDR correction. For all three SLDs, the highest ORs are found for ADHD and depression. The odds for depression appear comparable between the three SLD-domains (range 2.93–3.44). ADHD is more prevalent in cases of arithmetic [3.7 (95% CI = 2.61–5.28)] or spelling [3.81 (95% CI = 2.75–5.32)] disorder, compared to reading disorder [2.23 (95% CI = 1.57–3.24)].

### Relationship Between the Number of SLDs and the Risk for Psychopathology

The risk for psychopathology increases with increasing number of SLDs. The trend tests show a significant positive trend for the rates of all four disorders (anxiety disorder: z = 4.46, p < .001; depression: z = 9.76, p < .001; ADHD: z = 9.62, p < .001; conduct disorder: z = 5.45, p < .001) over the three levels “no SLD”, “isolated SLD”, and “comorbid SLD”. The estimates for the trend indicate that the odds of having depression or ADHD increase by a factor of 2.5 per level (depression: OR = 2.52; 95%-CI = 2.09–3.03; ADHD: OR = 2.51; 95%-CI = 2.08–3.03). The estimates for anxiety disorder (OR = 1.57; 95%-CI = 1.29–1.91) and conduct disorder (OR = 1.73; 95%-CI = 1.42–2.1) indicate an increase in odds by around 50% per level.

**Figure 3:**
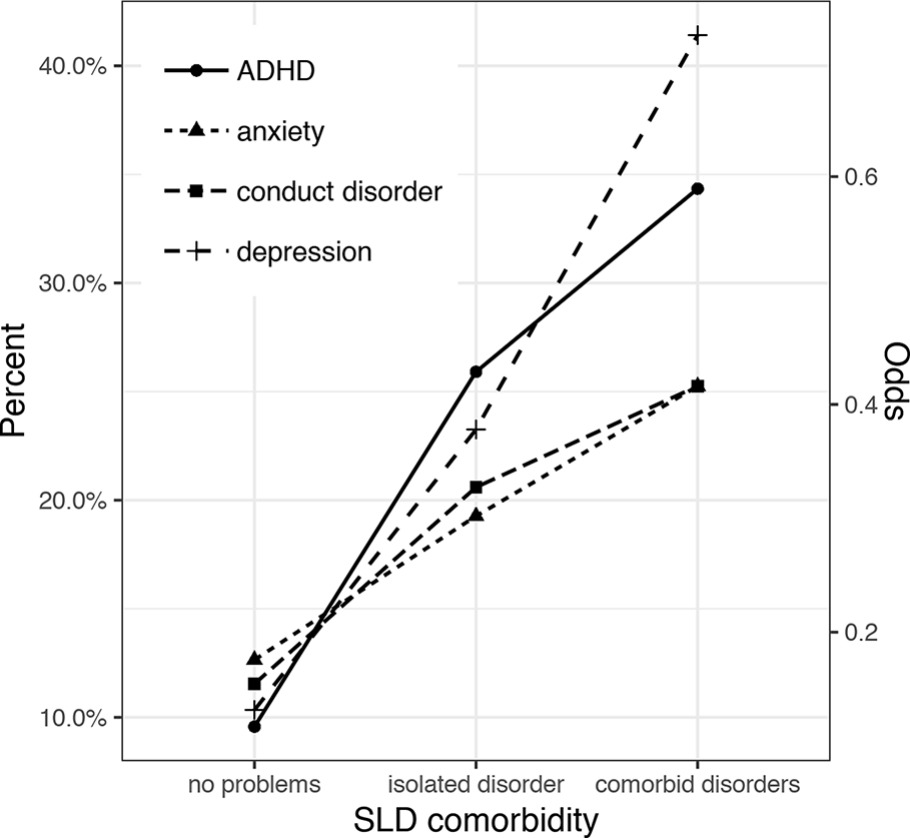
Trend in the prevalence of psychopathologies over the groups of children without a specific learning disorder (SLD), with an isolated SLD, and with comorbid SLDs.

The results of the post-hoc one-sided Fisher’s exact tests show that the occurrence of depression increases significantly over the three levels (no vs. isolated SLD: OR = 2.63, 95%-CI = 1.95–3.56, p < .001; isolated vs. comorbid SLD: OR = 2.33, 95%-CI = 1.45–3.74, p < .001). For the other three types of psychopathology, the trend is explained by a higher occurrence in children with an isolated SLD compared to no SLD (anxiety disorder: OR = 1.65, 95%-CI = 1.21–2.27, p = .002; ADHD: OR = 3.3, 95%-CI = 2.47–4.45, p < .001; conduct disorder: OR = 1.99, 95%-CI = 1.47–2.72, p < .001). The increase in occurrence from isolated SLD to comorbid SLDs is not significant (anxiety disorder: OR = 1.42, 95%-CI = 0.84–2.42, p = .13; ADHD: OR = 1.5, 95%-CI = 0.93–2.43, p = .069; conduct disorder: OR = 1.3, 95%-CI = 0.78–2.22, p = .201). **Figure 3** illustrates the increase in occurrence of the psychopathologies with the number of SLDs.

### Relationship Between the Number of SLDs and the Number of Psychopathologies

The Poisson regression model describing a child’s number of psychopathologies as a function of its number of SLDs shows a significant positive relationship between the two variables. The predicted number of psychopathologies for a child without SLD is about 0.5 [exponentiated intercept = 0.45 (95%-CI = 0.43–0.48, p < .001)]. The predicted number of psychopathologies increases by 66% when the number of SLDs increases by 1 (exponentiated slope = 1.66 [95%-CI = 1.55–1.79, p < .001)].

## Discussion

In the current study, we explored the occurrence of anxiety disorder, depression, ADHD, and conduct disorder in children with SLD in reading, spelling, and/or arithmetic skills. We inspected comorbidities between the different forms of psychopathology in children with SLD and differences between children with isolated vs. comorbid SLD in occurrence of psychopathology.

The results show that children with SLD more often had psychiatric disorders than children with no SLD. For children with any SLD the occurrence rates are 21% (anxiety disorder), 28% (depression), 28% (ADHD), and 22% (conduct disorder). The percentage of children with psychopathology in at least one area is clearly higher in children with an SLD than in those without an SLD. More specifically, and worryingly, while the chance of having psychopathology in one area is not significantly increased in children with an SLD, the chance of having psychopathology in two or more areas is. This comorbidity between the different types of psychopathology is also reflected in the relatively strong relationship we found between symptoms of anxiety disorder, depression, ADHD, and conduct disorder. ADHD appears more prevalent in children with arithmetic or spelling disorder, compared to reading disorder. Conduct disorder was not associated with isolated reading disorder. As hypothesized, children who have SLD in multiple domains have both a higher risk of having a psychiatric disorder and on average a higher number of psychiatric disorders.

The higher risk of internalizing and externalizing problems in children with SLD is in line with the results of earlier studies (e.g. 4). Whereas ADHD is often described as the most frequently occurring comorbidity in children with SLD (3) and we therefore expected to find the highest comorbidity rates with SLD, we found similarly high comorbidity rates for depression. The higher comorbidity rates for both ADHD and depression were also found when looking at the variables in a continuous manner (e.g., reflected in relatively high correlation coefficients with learning ability scores).

Our results show that it is important to distinguish between different types of SLD when studying differences in psychopathological symptoms. Also the distinction between reading and spelling disorder seems to be of importance, because they are associated with different comorbidity rates with psychopathological symptoms. More specifically, the prevalence of both ADHD and conduct disorder was higher in children with an isolated spelling disorder than in those with an isolated reading disorder. Furthermore, spelling disorder but not reading disorder showed a comorbidity with conduct disorder. More research is needed in order to explain these differences between isolated spelling and reading disorder. However, recent research has shown that reading and spelling disorder are related to distinct neurocognitive profiles (36). We speculate that these underlying differences in neurocognitive profiles result in behavioral differences and thereby explain the comorbidity between SLD and psychopathological symptoms.

Earlier research (4, 14) found an impact of ADHD on the relationship between SLD and other psychopathologies. In our study, we found that some children with psychopathological symptoms (e.g., conduct disorder) had comorbid ADHD, but others did not. ADHD does thus not always play a role in the comorbidity between SLD and conduct disorder. However, as we did not explicitly study moderation, we cannot rule out the existence of a moderating effect over the whole group. Contrary to Schuchardt et al. (5), but in agreement with Haberstroh and Schulte-Körne (13), we did find an increased occurrence of ADHD in children with arithmetic SLD. In fact, ADHD was more prevalent in children with isolated arithmetic disorder (25%) than in children with isolated reading disorder (16%) in our sample. The finding that SLD in multiple domains is related to higher rates of psychopathology is consistent with the results of previous research (4, 16).

The question remains how the comorbidity between SLD and psychopathology can be explained. Comorbidity of two or more disorders might have various causes. It might be due to direct or indirect causal relationships between two (or more) disorders or due to common underlying factors (37). A direct relationship between different disorders means that the likelihood for one disorder increases with the occurrence of another disorder [e.g., a diagnosis of a reading disorder increases the likelihood for the diagnosis of a spelling disorder; (38)]. An indirect causal relationship between two disorders exists when one disorder is associated to a third variable that in turn might evoke a second disorder. For example, SLD might lead to poor scholastic achievement and thereby elicit a depression.

Additionally, comorbid disorders might emerge due to common underlying factors such as common biological factors (37). An example of a common biological factor is shared genetic variance between different disorders. With regard to SLD, two studies showed a shared genetic variance in ADHD with reading and spelling disorder (39, 40). Possibly related to this, deficits in executive functions appear to be a key feature in both SLD and ADHD (4, 15, 41, 42). Furthermore, ADHD by itself has been suggested to be (partly) responsible for the comorbidity between SLD and other forms of psychopathology (4). Symptoms of inattention could be either a cause or a consequence of learning problems and might elicit psychopathological problems as well. At last, the exposure to different risk factors might evoke different disorders (such as a SLD and a depression) at the same time (43). However, more future research is needed to explain the underlying mechanisms of comorbidity between SLD and different mental disorders.

It is important to keep in mind that various factors contribute to the development and the progression of SLD. As underlined by the ecological systems theory (44), child development is affected by the various settings in which a child lives, the interactions between them, and external contexts, like the school system and culture. For example, genetic factors seem to play a more important role for the development of reading disorder in children with more educated parents than in children with less educated parents (45). This implies that in less educated families, environmental factors (such as lack of reading practice or poor instruction) play a more important role for the development of reading disorder than in higher educated families (45). Additionally, children may deal with their SLD in different ways. For example, children with a higher intrinsic motivation to ameliorate their SLD are more likely to take advantage of SLD assistance than children with lower intrinsic motivation. Emotional factors, such as a child’s self-efficacy and self-confidence, or the availability of adequate coping strategies to deal with scholastic setbacks due to SLD, may help to minimize the development of comorbid psychopathological symptoms. It can thus be assumed that the development of SLD and comorbid psychopathological symptoms are the result of complex interactions between genetic, environmental, and emotional factors.

### Limitations and Directions for Future Research

Even though our study had a large overall sample, the classification into the various groups resulted in relatively small group sizes. In the subsequent inferential statistical analysis, we used more general, and thus larger, classifications of SLDs to ensure sufficient power.

Mothers with a high educational level, which could hint at high SES, were overrepresented in our sample. Both dyslexia (46) and underachievement in math (47) might be more prevalent in children from low SES-families. Low parental education is also related to higher degrees of anxiety disorder and depression in children (48) and may constitute a risk factor for children to develop ADHD (49, 50) as well as conduct problems (51). As SES seems related to both learning disorders and psychopathology, the overrepresentation of mothers with high educational background could have yielded an underestimation of learning disabilities and psychopathological symptoms in our results. We perceive this possible influence as relatively unproblematic, as we aimed to study not prevalence rates but the relationship between SLD and psychopathology. To our knowledge, no research has yet been done on the influence of SES on the relationship between SLD and psychopathology, which we consider to be an important question for future research.

Another point to keep in mind is the fact that we presented tests and questionnaires in a web-based application. Although the content is the same as in the original paper-pencil-versions, the validity of the online instruments and possible differences between writing and typing is still focus of ongoing research. Also, we did not have the possibility to observe the test administrations. This means that we cannot be completely sure that the children were not helped by others while working on the tests, for example. We have excluded unreliable data as far as that was possible (e.g., when patterns suggesting unreliability could be observed in the data) on the basis of the plausibility checks. The online format can be seen as a strength of the study as well, as it has made it possible to reach a large sample size and to include the motivational concept.

Also, the standardized test results have been based on norms that we developed based on the sample of the current study. This means that the frequency of the SLDs and psychopathology in the total sample is not informative, as it is the pure consequence of the norming process. However, our study focuses on the comorbidity between SLDs and psychopathology, which can be well studied using norms based on the study sample.

In the current study, the choice for cut-offs to classify children as having or not having a specific SLD or psychopathology are not the same for SLD and psychopathology, but they follow the guidelines as set by the DSM-5 and the DISYPS-II, respectively. This means they conform with the criteria as they are used in daily practice. However, the use of cut-offs may have influenced our results. To avoid this influence, future research could analyze the relation between SLDs and psychopathology in a continuous manner. In addition, the identification of subgroups of children with specific combinations of SLDs and psychopathology could be a topic for future research. Future research using longitudinal designs is needed to identify the causal pathways leading to the comorbidities, which are still largely unknown (1). In addition, because of the relatively high comorbidity rate not only with psychopathology, but also between different SLDs, a relevant question for future research would be if domain-specific or cross-domain learning interventions are more effective.

### Implications for Practice

Knowledge about the comorbidity between SLDs and anxiety disorder, depression, ADHD, and conduct disorder has important implications for the support of children with SLD in daily praxis. For example, our results mean that children who are suspected or known to have an SLD should especially be screened for symptoms of depression and ADHD, even more so with learning difficulties in multiple domains. Teachers need to be trained in noticing learning as well as psychopathological problems in children in an early stage, so that intervention can prevent more severe problems. In addition, psychopathology should be taken into account when planning a learning intervention, because they might interfere with the effectiveness of the intervention, which is highest when optimally tailored to the child (52).

To give an example, a depressive mood often manifests in feelings of inferiority, little self-efficacy and general listlessness. Children who are suffering from depression might not attend reading, spelling and arithmetic interventions as they have the feeling that they do not succeed anyway. On the other hand, earning bad marks in school due to their SLD may constitute a further mental burden and reinforce the depressive mood. Special interventions addressed to children with both SLD and depression should focus on improving self-efficacy.

Attention problems, impulsivity, and hyperactivity might also hinder the effectiveness of learning interventions. Children with ADHD often have difficulties to focus on quiet activities, especially when they know they are not good at them (e.g., in the area of their SLD). Positive reinforcement of the child is of great importance, e.g. in the form of token systems in SLD interventions with children with ADHD. In summary, in both depression and ADHD, the interaction of psychotherapeutic methods and SLD intervention is imperative.

## Conclusions

Depression and ADHD, and to a lesser extent anxiety disorder and conduct disorder, are elevated in children with SLD in reading, spelling, and/or arithmetic skills. In children with SLD in multiple learning domains both the chance of psychopathology and the number of psychopathological areas are higher than in children with an isolated SLD. These findings underline the relevance of detecting psychiatric comorbidities in children with SLD in order to provide the best possible support to affected children. Possibilities to implement psychotherapeutic methods in interventions for SLD are discussed.

## Data Availability Statement

All datasets generated for this study are included in the manuscript/supplementary files. The data and analysis code for this study can be found in the Open Science Framework (https://osf.io/9mxp2/).

## Ethics Statement

The studies involving human participants were reviewed and approved by Ethics committees of the University Hospital of the Ludwig-Maximilians-University Munich and of the DIPF | Leibniz Institute for Research and Information in Education, Frankfurt am Main. Written informed consent to participate in this study was provided by the participants’ legal guardian/next of kin.

## Author Contributions

All authors contributed to the preparation of the study and the data collection. JL and LV performed the data preparation. JL wrote the scripts for and executed the statistical analyses. JK and LV wrote the first draft of the manuscript. JL and RG wrote sections of the manuscript. All authors read and approved the submitted version.

## Funding

This work was supported by the German Federal Ministry of Education and Research (BMBF) [grant numbers 01GJ1601A and 01GJ1601B].

## Conflict of Interest

The authors declare that the research was conducted in the absence of any commercial or financial relationships that could be construed as a potential conflict of interest.

The reviewer SS declared a shared affiliation, with no collaboration, with several of the authors, MH and GS-K, to the handling editor.

## Footnotes

1 As we developed the norms on the basis of the current study’s sample and we defined an SLD as a z-score of -1.5 or lower, the percentage of children with a specific type of SLD (e.g., a reading disorder) will per definition be the percentile rank that corresponds to a z-score of −1.5, which is 6.7%.
