# Supplementary material for: Comorbidities Between Specific Learning Disorders and Psychopathology in Elementary School Children in Germany
Source: Front Psychiatry. 2020 Apr 28;11:292. doi: 10.3389/fpsyt.2020.00292 (PMC7198840; doi:10.3389/fpsyt.2020.00292)
Supplement: Supplementary file 1 [file DataSheet_1.docx]

Supplementary Material

# Implausible Data

We applied five plausibility checks to the data, for which we determined the necessity and exact criteria based on the dataset itself.

Firstly, we checked whether the total time that a child worked on a specific test deviated from the set maximum time limit for this test by more than an acceptable tolerance limit. The tolerance limits were chosen on a test-by-test basis for positive and negative deviations, respectively: CFT 20-R (30 sec and 6 sec); CODY-M 2-4 (15 sec and 1 sec); WLLP-R (4 sec and 1 sec). Test results from children whose testing time exceeded these limits were considered implausible and excluded from further analysis.

Secondly, we checked whether a child selected the same answer alternative in the WLLP-R repetitively for an unrealistic number of times. Test results from children who exceeded 10 consecutive repetitions of the same answer alternative were considered implausible and excluded.

Thirdly, we checked whether a child responded unrealistically quickly, i.e., if it just typed a key instead of seriously working on the test. For each instrument, we first computed the median of the distribution of all single trial response times over all participants. For each response time in the left tail of this distribution (response times that are smaller, i.e., faster, than the median), we then computed the absolute deviation from the median and standardized it by dividing it by the median absolute deviation (MAD; i.e., the median of the absolute deviation from the median). We considered absolute deviations larger than three times the MAD as unrealistically quick and excluded test data from children who had implausibly fast response times for a certain percentage of all items of the respective tests. For most tests this criterion was set to 15%; for the CODY-M 2-4 subtests ‘missing number’ and ‘domino count comparison’, it was set to 30%. For the subtest ‘domino count comparison’, we additionally only considered test results as implausible if less than 70% of the items were answered accurately, because most of its items were very easy so that very fast reaction times were to be expected.

Fourthly, we checked whether participants seemed to have typed random letters instead of words when they had to fill in the blanks in the spelling tests WRT3+ and WRT4+. For each typed answer, we determined the similarity to its target word by computing the Jaro-Winkler distance (Jaro, 1989; Winkler, 1990). Answers with a Jaro-Winkler distance of more than .41 (.49 for the German word ‘Axt’) were considered as random typing. Test results from children who typed randomly for more than 15% of the items were considered implausible and excluded.

Fifthly, and finally, we excluded test results for the CFT 20-R and the CODY-M 2-4 if a participant did not complete all subtests.

In total, we excluded 540 (11.9%) cases because of implausible data. No data of parent questionnaires appeared to be implausible.

# Standardization

We decided to compute norms based on our own sample instead of using the norms from the norm sample of the original tests as a) the testing conditions in the web-based application deviated from the original tests (mostly paper-pencil versions), b) normalizing the tests to the same sample yields a higher degree of comparability between the test results, and c) our sample was considerably larger than the norm samples of the different tests, resulting in more precise norms.

The norms for the CFT 20-R, CODY-M 2-4, WRT 3+ and 4+, and WLLP-R were developed separately for the 3rd and 4th grade. For the SCARED-D and DISYPS-II, the norms were developed for girls and boys separately, which is in line with the norms of the original DISYPS-II.

Before normalizing children’s psychopathology to our sample, we compared the occurrence of psychopathology in our sample based on the norms from the original tests, to the results from the BELLA study (Ravens-Sieberer et al., 2008), a prevalence study of mental health problems in children and adolescents in Germany. The differences were mostly small and might be attributed to the different methods used to assess psychopathology in the two studies. However, the occurrence of conduct disorder was lower in our sample. As conduct disorder is more prevalent in children from families with a low SES background (Matthys & Lochman, 2017), this difference might be attributed to the overrepresentation of mothers with high educational level in our study.

Table

*Average Test Scores for the Different SLD Groups*

| SLD group | CFT | | WRT | | WLLP-R | | CODY | |
| --- | --- | --- | --- | --- | --- | --- | --- | --- |
|  | (intelligence) | | (spelling) | | (reading) | | (arithmetic) | |
|  | [IQ] | | [T-score] | | [T-score] | | [T-score] | |
|  | M | SD | M | SD | M | SD | M | SD |
| no disorder | 103.9 | 13.7 | 53.7 | 7.7 | 54 | 7.6 | 53.8 | 7.6 |
| isolated reading disorder | 101.7 | 12.7 | 49.7 | 6.8 | 32 | 2.1 | 49.7 | 6.5 |
| isolated spelling disorder | 98.3 | 12.4 | 31.9 | 2.2 | 48.2 | 5.6 | 51 | 8.1 |
| isolated arithmetic disorder | 93.1 | 11.4 | 48.9 | 7.1 | 47.7 | 6.4 | 31.9 | 2 |
| comorbid reading & spelling | 96.9 | 16.3 | 31 | 2.2 | 30.9 | 2.1 | 47.3 | 5.2 |
| comorbid reading & arithmetic | 92.6 | 10.1 | 45.1 | 4.1 | 32.8 | 2.2 | 31.3 | 2.3 |
| comorbid spelling & arithmetic | 89.5 | 11.1 | 33 | 2.1 | 46.6 | 4.2 | 30.4 | 2.1 |
| comorbid reading, spelling, & arithmetic | 88.9 | 10.3 | 30.6 | 2.1 | 30.7 | 2.2 | 30.9 | 1.6 |

*Note.*The average intelligence quotients as well as reading, spelling, and arithmetic T-scores. The average IQ is lower for children with an isolated arithmetic disorder compared to an isolated reading or spelling disorder (M = 93.11 [95% CI = 90.06–96.15] vs. M = 101.73 [95% CI = 98.3–105.16] respectively M = 93.11 [95% CI = 90.06–96.15] vs. M = 98.3 [95% CI = 94.67–101.92]). This could be related to the fact that children with dyscalculia are known to have difficulties in visual-spatial processing (Mähler & Schuchardt, 2012) and executive functions (especially inhibition of information; Deutsche Gesellschaft für Kinder- und Jugendpsychiatrie, Psychosomatik und Psychotherapie, 2018). The nonverbal intelligence test used, in significant part, draws upon these skills, which might have adversely affected the performance of children with dyscalculia.
